# Enhanced offspring predisposition to steatohepatitis with maternal high-fat diet is associated with epigenetic and microbiome alterations

**DOI:** 10.1371/journal.pone.0175675

**Published:** 2017-04-17

**Authors:** Umesh D. Wankhade, Ying Zhong, Ping Kang, Maria Alfaro, Sree V. Chintapalli, Keshari M. Thakali, Kartik Shankar

**Affiliations:** 1 Arkansas Children’s Nutrition Center, Little Rock, Arkansas, United States of America; 2 Department of Pediatrics, University of Arkansas for Medical Sciences, Little Rock, Arkansas, United States of America; 3 Molecular Genetic Pathology Laboratory, Arkansas Children’s Hospital, Little Rock, Arkansas, United States of America; The University of Manchester, UNITED KINGDOM

## Abstract

**Objective:**

Non-alcoholic fatty liver disease (NAFLD) is an important co-morbidity associated with obesity and a precursor to steatohepatitis. However, the contributions of gestational and early life influences on development of NAFLD and NASH remain poorly appreciated.

**Methods:**

Two independent studies were performed to examine whether maternal over-nutrition via exposure to high fat diet (HFD) leads to exacerbated hepatic responses to post-natal HFD and methionine choline deficient (MCD) diets in the offspring. Offspring of both control diet- and HFD-fed dams were weaned onto control and HFD, creating four groups.

**Results:**

When compared to their control diet-fed littermates, offspring of HF-dams weaned onto HFD gained greater body weight; had increased relative liver weight and showed hepatic steatosis and inflammation. Similarly, this group revealed significantly greater immune response and pro-fibrogenic gene expression via RNA-seq. In parallel, 7–8 week old offspring were challenged with either control or MCD diets for 3 weeks. Responses to MCD diets were also exacerbated due to maternal HFD as seen by gene expression of classical pro-fibrogenic genes. Quantitative genome-scale DNA methylation analysis of over 1 million CpGs showed persistent epigenetic changes in key genes in tissue development and metabolism (*Fgf21*, *Ppargc1β*) with maternal HFD and in cell adhesion and communication (*VWF*, *Ephb2*) in the combination of maternal HFD and offspring MCD diets. Maternal HFD also influenced gut microbiome profiles in offspring leading to a decrease in α-diversity. Linear regression analysis revealed association between serum ALT levels and *Coprococcus*, *Coriobacteriacae*, *Helicobacterioceae* and *Allobaculum*.

**Conclusion:**

Our findings indicate that maternal HFD detrimentally alters epigenetic and gut microbiome pathways to favor development of fatty liver disease and its progressive sequelae.

## Introduction

The steep rise in obesity over the last quarter century has been mirrored by an increasingly troubling trend of obesity during pregnancy. At present, over 60% of all pregnancies in the United States are in women who are either overweight or obese at conception [1]. This is significant since maternal obesity confers several detrimental risk factors to the offspring, including the increased predisposition to obesity and metabolic dysfunction in adulthood [2]. Previous studies in animal models and clinical studies point to a role for maternal obesity in long-term programming of metabolic dysfunction [3,4]. In concert with other metabolic comorbidities, human obesity is strongly associated with non-alcoholic fatty liver disease (NAFLD) and steatohepatitis (NASH), which collectively represent the most prominent burgeoning challenge in liver disease.

In the United States, NAFLD is a leading cause of chronic liver disease including progression to NASH. While accumulation of ectopic lipids in the liver is a hallmark of NAFLD, the contributory mechanisms are complex and multi-factorial. An abundance of energy surplus accompanied by reduced physical activity and a sedentary lifestyle partially account for the etiology of NAFLD [5]. Since an increasing body of evidence points to the gestational origins of obesity and metabolic dysfunction via developmental programming, it is intuitive that the *in utero* environment may also affect susceptibility to metabolic co-morbidities such as NAFLD and NASH. Indeed, studies from our group and others have shown that maternal over-nutrition alters systemic metabolic and endocrine signaling, insulin sensitivity and reprograms lipid biosynthesis and oxidative pathways in the liver leading to hepatic steatosis [3,6,7,8,9,10]. Moreover, maternal high-fat diet is associated with enhanced fatty liver in fetuses and offspring in a non-human primate model [11]. Notably, infants of obese mothers also show greater intrahepatic lipid levels as assessed by MRI imaging soon after birth [12]. Despite these important studies, whether maternal diet and obesity alter development of other pathological features associated with fatty liver disease remains to be clarified. More importantly, specific mechanisms through which gestational and early-life exposure to maternal obesity predispose offspring to liver disease remain unanswered.

In the present study, we examine the hypothesis that maternal over-nutrition via *in utero* exposure to high fat diet (HFD) leads to developmental programming of obesity, and hepatic inflammatory and fibrogenic signaling in the offspring. To this end, we characterized offspring from lean and obese dams following postnatal challenge with either a) HFD or b) methionine choline deficient (MCD) diets in separate studies. We conducted analysis of offspring weight, body composition, and hepatic histology in response to these insults. Global changes in transcriptomic profiles were assessed using RNA-seq and confirmation was carried out using real-time RT-PCR. In order to further elucidate underlying pathways, we comprehensively examined two broad mechanisms. First, we investigated quantitative changes in DNA methylation in offspring liver at over 1 million CpGs using reduced representation bisulfite sequencing (RRBS). Second, we conducted global profiling of the offspring cecal microbiome using high-throughput sequencing of 16S rDNA amplicons. Our studies provide unique mechanistic insight into how maternal obesity during gestation and lactation alters epigenetic and gut microbiome pathways in a manner hypothesized to favor development of fatty liver and inflammation.

## Materials and methods

### Experimental design

Five week old female C57BL6/J mice (stock 0664, Jackson Laboratories, Bar Harbor, ME) were given *ad libitum* access to control (17% fat Harlan Teklad TD95095, n = 10) or high-fat diets (HF, 45% fat, TD08811, n = 10) for 12 weeks. At 17 weeks of age females were bred with lean male mice (fed control diets TD8640 throughout). Body weights were monitored weekly throughout and body composition was assessed non-invasively via QMR (EchoMRI) at 5 and 12 weeks of age. Upon birth, all offspring remained with birth dams until weaning and litter sizes were adjusted to 6 pups per litter. Offspring from control or HFD-fed dams were given access to control or HFD which led to four groups of offspring: viz. offspring born to control diet fed dams weaned onto Con (**CC**, n = 10) or HFD (**CH**, n = 11) and offspring born to HFD-dams weaned onto Con (**HC**, n = 7) or HFD (**HH**, n = 7) (S1A Fig). Post-weaning diet intervention was 14 weeks. Throughout the study, offspring body weight was monitored weekly.

In a separate study, seven-week old offspring from control and HF dams received control diet from weaning to 7 wk of age, at which time offspring from both dam groups were randomized to either a methionine choline deficient (MCD) diet (MP Biomedicals) (referred to as **C-MCD**, n = 12 or **H-MCD**, n = 9; from lean and obese dams, respectively) or equivalent control diet with similar macronutrient composition with methionine and choline (MP Biomedicals, cat# 960441)(referred to as **CC**, n = 11 or **HC**, n = 9; from lean and obese dams, respectively). Diet interventions with MCD and control diets lasted for 25 days. During the study, offspring body weight was monitored every three days. Offspring body composition was assessed at the start and at the end of the study (S2 Fig). Both male and female offspring were studied separately. However, since phenotypes of liver pathology (reported herein) were most prominent in males, only data from male offspring are presented. A separate report on comparisons between male and female offspring is in consideration elsewhere. Offspring were euthanized with CO_2_. Blood was collected via cardiac puncture for serum separation to measure NEFA, triglycerides, cholesterol and glucose. The liver was weighed, fixed in formalin or snap frozen in liquid nitrogen for subsequent RNA and protein analysis. Cecum was collected with intact contents and frozen for further analysis. The Institutional Animal Care and Use Committee at the University of Arkansas for Medical Sciences approved all experimental protocols.

### Histological analysis

Offspring livers were fixed in formalin and paraffin embedded before sectioning. One 6 μm section from each liver was stained with either hematoxylin and eosin or Picro-Sirius Red to assess the general histological features, extent of steatosis, inflammation and fibrosis using the Kleiner scoring system in a blinded fashion [13].

### Blood biochemistry and cytokine assessment

Serum glucose and triglycerides were assayed using colorimetric reagents (Fisher Scientific, Waltham, MA). Non-esterified fatty acids (NEFA) were quantitated with non-esterified fatty acid-C reagents (Wako Chemicals USA, Richmond, VA) [3]. Circulating cytokines were assayed using the V-PLEX Plus pro-inflammatory multiplex panel-1 covering 10 cytokines (Meso Scale Discovery).

### Real-time RT-PCR

Total RNA was isolated from liver using RNeasy mini columns (QIAGEN, Valencia, CA) including on-column DNase digestion. One microgram of total RNA was reverse transcribed using the iScript cDNA synthesis kit (BioRad, Hercules, CA). Realtime PCR analysis was performed as described previously using an ABI Prism 7500 Fast instrument (Carlsbad, CA) [6]. Gene specific primers were designed using Primer Express Software (S1 Table). Relative amounts of mRNA were quantified using a standard curve and normalized to the expression of SRP14 or cyclophilin A mRNA.

### Preparation of RNA-seq libraries and data analysis

RNA-seq was performed on livers from CC, CH, HC and HH groups at 20 weeks of age. RNA was isolated from livers of individual offspring (n = 7–12). cDNA libraries were prepared using poly-A mRNA from each individual RNA sample (**Supplementary Methods** for details) [14]. Equal amounts of polyA-mRNA from 2–3 mice were pooled, to generate three biologically distinct replicates per group representing all animals (CC n = 10, CH n = 11, HC n = 7, HH n = 7). Single read 75-bp sequencing of libraries was performed using a NextSeq500 (Illumina). Data analysis was carried out using the Tuxedo pipeline (TopHat-Cufflinks-CummeRbund) and visualized in SeqMonk [15]. The lists of differentially expressed genes were analyzed for GO biological process and molecular function enrichment using the BiNGO and ClueGO plugins in Cytoscape. The aligned sequencing data are available at the NCBI SRA archive as Bioproject PRJNA380108.

### Genome-scale DNA methylation via RRBS

DNA methylation changes associated with maternal HFD and offspring MCD challenge were assessed using RRBS, which involves sequencing of bisulfite-converted *MspI* fragment libraries. Liver genomic DNA was isolated using a combination of proteinase-K digestion and Purelink genomic DNA isolation kits (Life Technologies). Three biologically distinct pools of genomic DNA (containing n = 3 in each pool) were utilized to generate libraries. RRBS libraries were prepared as described by Gu *et al* [16] with detailed methods provided in Supplementary methods. Reads were trimmed for adapter sequences using Trim Galore and filtered for quality score. Alignment and methylation calling were performed using Bismark. We first examined whether maternal HFD and offspring diets affected methylation of promoters and CpG islands (CGI). Promoters were sub-classified into those overlapping or devoid of CGIs. Frequency distribution of methylation status of features was computed. Statistical differences between groups were analyzed using χ^2^ test. Data analysis and summarization were done using SeqMonk and the DSS package in R [17]. Comparisons between different groups (CC, C-MCD, HC and H-MCD) were performed using Wald test, and *P* values were adjusted for multiple testing using the FDR method. CpGs with *p* < 0.0001 and a minimum difference in methylation (Δ_me_) of 10% were retained. Differentially methylated regions (DMRs) were annotated with the closest/overlapping transcription start sites (TSSs) (±10 kb). The lists of differentially expressed genes were analyzed for GO biological process and molecular function enrichment using the BiNGO plugin in Cytoscape.

### Microbial community profiling via 16S rRNA amplicon sequencing

Bacterial DNA was isolated from cecal contents using QIAamp Fast DNA stool mini kit (Qiagen) including a bead-beating step. Fifty nanograms of genomic DNA was utilized for amplification of the V4 variable region of the 16S rRNA gene using 515F/806R primers. Forward and reverse primers were barcoded to accommodate multiplexing up to 384 samples per run as described by Kozich et al [18]. Paired-end sequencing (2 X 250 bp) of pooled amplicons was carried out using Illumina Miseq platform with ~30% PhiX DNA.

Processing and quality filtering of reads was performed by using scripts in QIIME (v1.9.1) [19]. OTU picking was performed using an open-reference method and representative sequences were further aligned using PyNAST with the Greengenes core-set alignment template. Construction of the phylogenetic tree was performed using QIIME. Alpha rarefaction was performed using the phylogenetic diversity, Chao1 and observed species metrics. *β*-diversity estimation was carried out by computing weighted and un-weighted UniFrac distances between samples using QIIME. Differences in OTU abundance between groups were identified using STAMP [20] and visualized using Clustvis. PICRUSt was used to identify differences in predictive metagenome function [21]. We also examined group difference using LefSe which utilizes Linear Discriminant Analysis of Effect Size. Associations of OTU abundance with serum ALT levels were performed using MaAsLin which is a multivariate statistical framework that performs boosted, additive general linear models. Analysis for LefSe and MaAsLin were carried out using the default settings [22].

### Statistical analysis

Data are expressed as means ± SEM. Statistical analyses were performed using Graphpad Prism version 6, R-Bioconductor and QIIME. Description of statistical analysis of RNA-seq, DNA methylation and microbiome is presented in the respective sections. Statistical significance was set at p ≤ 0.05. Maternal variables including body weight, and body adiposity were assessed via two-tailed Students t-test. Offspring body weight, body composition, and hepatic gene expression data were analyzed by using one-way ANOVA and two way ANOVA where appropriate. Two-way ANOVA was employed to determine the main effects of maternal diet, offspring diet and interaction thereof. Significant interactions identified by two-way ANOVA were followed by a one-way ANOVA and all pair-wise comparisons by Student-Newman-Keuls post hoc tests.

## Results

### Maternal HFD is associated with greater weight gain, fatty liver and programing of a pro-inflammatory hepatic transcriptome in offspring

**Fig 1:**
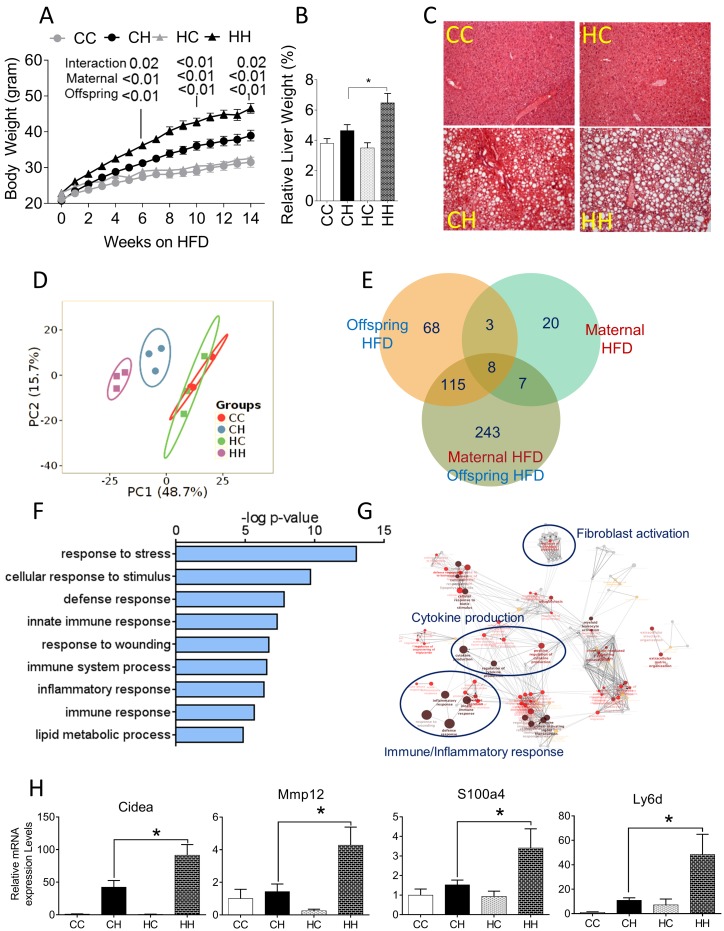
Offspring characteristics and hepatic transcriptome analysis. **(A)** Body weight and **(B)** Relative liver weights of offspring from control and HFD-fed dams weaned onto either Con (**CC**, n = 10; **HC**, n = 7) or HFD (**CH**, n = 11; **HH**, n = 7). Offspring were provided *ad libitum* access to Con or HF diet for 14 wk after weaning. **(C)** Photomicrographs of H&E stained liver sections from offspring. **(D)** Principal component analysis of global gene expression profiles showing unsupervised clustering of samples based on maternal and post-weaning HFD. **(E)** Venn diagram showing the number of differentially expressed transcripts due to maternal and offspring HFD (± 2.0-fold change, p<0.05, corrected for multiple testing). **(F)** Analysis of GO biological process terms via BiNGO and **(G)** Clustering using ClueGO, among differentially expressed genes showing enrichment of immune response, inflammatory pathways and fibroblasts activation. **(H)** Real-time RT-PCR based confirmation of mRNA expression of genes differentially expressed in the combination of maternal obesity and offspring HFD. Data are expressed as means ± SE. Statistical differences in body weight were determined using two-way ANOVA to examine the main effects of maternal and post-weaning HFD diet, followed by Student-Newman-Keuls *post hoc* analyses. Statistical differences in gene expression were determined using a one-way ANOVA. (*p<0.05, **p<0.01).

Dams on HFD gained significantly more weight and had a higher percentage of body fat compared to their control diet-fed counterparts (S1B and S1C Fig). Overall litter sizes, as well as offspring sex ratio were not significantly different between HFD and Con diet fed groups (S1D Fig). Despite no differences in birth weight, male pups born to HFD fed dams showed greater body weight at weaning suggesting greater weight gain during lactation (S1E Fig). As anticipated, offspring weaned to a HFD gained more weight over the post-weaning period compared to littermates fed control diet (Fig 1A). However, offspring from HFD-fed dams showed distinct hyper-responsiveness to post-weaning HFD, as evidenced by greater weight gain, and fat mass relative to offspring from control-diet dams (S1A and S1F Fig). Two-way ANOVA showed that as anticipated offspring diet has a profound effect on offspring body composition. Additionally, there was significant interaction between maternal obesity and offspring diet. Finally, exposure to maternal obesity significantly increased fat mass in the offspring from obese dams relative to offspring from control diet-fed dams (S1F Fig). The combination of maternal and offspring HFD also led to a significant increase in relative liver weights (p<0.01) (Fig 1B). Histological analysis of hematoxylin and eosin stained liver sections confirmed prominent fatty liver in both HFD fed groups (CH, HH) (Fig 1C). However offspring of HFD-fed dams on HFD (HH offspring), showed significantly greater parenchymal area with steatosis and prominent immune cell infiltration (Fig 1C). Analysis of circulating serum metabolites showed cholesterol (p<0.001) and NEFA (p<0.05) were significantly affected by maternal HFD and combination of maternal and offspring HFD, respectively. Glucose and triglycerides were not different between the groups (S2 Table).

RNA-seq analysis was performed to ascertain global changes in transcriptional profiles. De-multiplexed reads for each biological replicate resulted in average of 31 million reads. Alignment showed that the majority of reads (>91%) in all samples mapped to exons and approximately 9% mapped to intronic regions. To assess global gene expression profiles, read counts were converted into RPKM. The range and distribution of RPKM were similar for all samples, suggesting comparable transcriptomic coverage. Principal component analysis of expression data showed grouping based post-weaning HFD challenge. Further, within HFD fed offspring, offspring from control and HFD-fed dams clustered separately, showing a robust effect of maternal HFD (Fig 1D).

Maternal HFD and offspring HFD *per se*, altered the expression of 20 and 68 transcripts, respectively (minimum ± 2.0-fold change, adjusted *P*<0.05 corrected for multiple testing). However, the combination of maternal and offspring HFD together led to differential expression of 243 unique transcripts (Fig 1E, S3 Table). Enrichment of GO biological process terms revealed that genes involved in inflammatory pathways such as innate immune response, immune system processes and lipid metabolic processes were up-regulated due to the combination of maternal and offspring HFD (Fig 1F). Consistent with GO enrichment analysis, ClueGO a clustering algorithm, also highlighted inflammatory pathways such as cytokine production, fibroblast activation and immune/inflammatory processes to be upregulated by the combination of maternal and offspring HFD (Fig 1G). Real-time RT-PCR was used to confirm many of the targets identified by RNA-seq. Genes involved in fibrosis (*Cidea*, *Mmp12*), and inflammation (*Ly6d*) were significantly up-regulated in livers of HH compared to CH groups (p<0.05, Fig 1H, S4 Table). Overall, exposure to maternal HFD clearly influenced hepatic gene expression of inflammatory and fibrogenic pathways.

### Maternal HFD exacerbates hepatic pro-fibrogenic response to a post-weaning MCD diet

**Fig 2:**
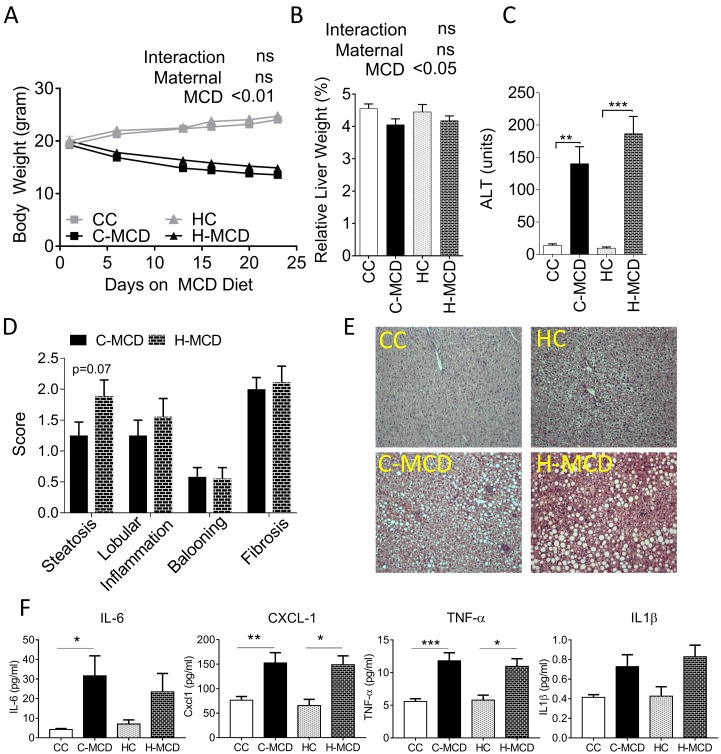
Effect of maternal HFD on offspring response to MCD diet challenge. **(A)** Body weight of offspring from control and HFD-fed dams. Offspring were provided either Con diet (**CC**, n = 11 or **HC**, n = 11) or MCD (**C-MCD**, n = 9 or **H-MCD**, n = 9) for 25 days starting at 7 wk of age. **(B)** Relative liver weights and **(C)** Serum alanine aminotransferase (ALT) concentrations in offspring. **(D)** Kleiner scores for steatosis, lobular inflammation, ballooning and fibrosis compared between C-MCD and H-MCD groups. **(E)** Photomicrographs of H&E stained liver sections from offspring. Data are expressed as means ± SE. Statistical differences in body weight were determined using two-way ANOVA to examine the main effects of maternal and post-weaning MCD diet, followed by Student-Newman-Keuls *post hoc* analyses. Statistical differences pathology scores were assessed using Students t-test. (*p<0.05, **p<0.01, ***p<0.001).

**Fig 3:**
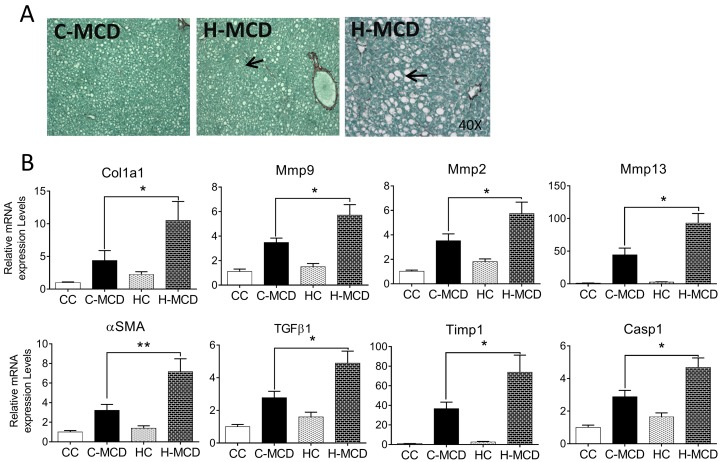
Maternal HFD increases pro-fibrogenic response in offspring liver following MCD diets. **(A)** Photomicrographs of Picro-Sirius red stain showing peri-cellular fibrosis. **(B)** Real-time PCR quantification of the genes involved in fibrosis, steatosis and inflammation (*Col1a1*, *Mmp9*, *Mmp2*, *α-SMA*, *Mmp13*, *Timp1*, *Casp1 and Tgfβ1*). Data are expressed as means ± SE. Statistical differences in gene expression were determined using a 2-way ANOVA to examine the main effects of maternal HFD and offspring MCD diet, followed by Student-Newman-Keuls post hoc analyses (*p<0.05, **p<0.01).

Offspring from control and HFD fed dams were provided MCD or an appropriate control diet to examine inflammatory changes in the absence of HFD (S2 Fig, referred to as CC, C-MCD, HC or H-MCD groups). As anticipated, MCD diets induced significant weight loss and severe hepatic inflammation (p<0.001) (Fig 2A and 2B) in both sets of offspring (C-MCD and H-MCD). Two-way ANOVA showed that offspring MCD diet has a profound effect on relative liver weights. Relative liver weights were significantly reduced in MCD challenged groups irrespective of maternal HFD (MCD p<0.001). However relative liver weights, extent of liver injury assessed via serum ALT and qualitative histological scoring were not different between the two MCD-diet groups (Fig 2B–2D). Serum ALT and steatosis scores of livers though numerically greater in H-MCD offspring, did not reach statistical significance. This was also reflected in larger lipid droplets and greater presence of pyknotic nuclei in H&E stained sections (Fig 2E). Circulating levels of pro-inflammatory factors (TNF-*α*, IL-6 and CXCL-1, p<0.05) also did not differ between MCD-fed offspring of lean and HFD-dams (Fig 2F, data for other cytokines in S6 Table). Nonetheless, Picro-Sirius Red stained sections showed greater extent of peri-cellular fibrosis in H-MCD offspring relative to C-MCD offspring (Fig 3A). mRNA expression of classical genes involved in apoptosis and fibrosis including *Col1a1*, *Mmp9*, *Mmp2*, *α-SMA*, *Mmp13*, *Timp1*, *Casp1* and *Tgfβ1*, were up-regulated in livers from offspring of HFD-dams compared to control dams (p<0.05). Two-way ANOVA revealed that maternal HFD (p<0.05) exacerbated the hepatic pro-fibrogenic gene expression response to a post weaning MCD diet (Fig 3B, S5 Table).

### Maternal HFD in combination with post weaning MCD diet triggers persistent changes in hepatic DNA methylation

**Fig 4:**
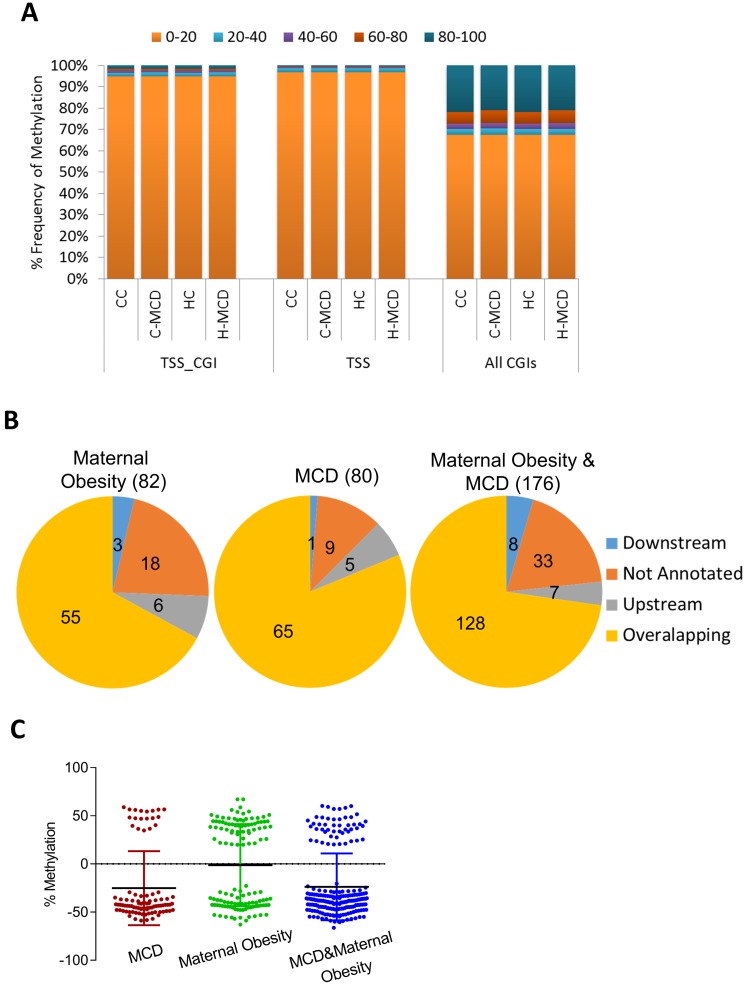
DNA methylation changes in livers of offspring from lean and obese dams challenged with MCD diet. Genome-scale DNA methylation was assessed using RRBS. **(A)** Percent frequency distribution of methylation status of informative promoters (TSS), promoters containing CGI (TSS_CGI), all CGIs, in offspring from lean and HFD dams challenged with control or MCD diet. Methylation status of features is binned into 5 categories (0%–20%, 20%–40%, and so on). **(B)** Genomic localization of differentially methylated regions (DMRs). **(C)** Δ_me_ (difference in average methylation between groups) for each DMR showing both hypo and hypermethylated regions. See full list in Supplementary tables. Each group was compared against CC offspring.

We employed RRBS to examine changes in DNA methylation with maternal and offspring (MCD) diets. RRBS quantitatively assayed methylation status of ~1.5 million CpGs (5X coverage) at base-pair resolution. Bisulfite conversion efficiency in all samples was >99.5%. We computed methylation of 33,764 promoters containing CGIs (±3 Kb from TSS), 24,572 CpG islands and 9,935 CGI within promoters (minimum 5 CpGs per feature). As anticipated, most CGIs (85%) and CGI promoters (90%) showed very low methylation (0%–20%). Maternal HFD or offspring MCD diet did not alter distribution of methylation in CGIs or CGI promoters (Fig 4A). Further analysis using the DSS package identified 82 DMRs associated with maternal HFD and 80 DMRs with offspring MCD diet, respectively (p<0.001; Δ_me_ 10%). The combination of maternal HFD and MCD diet had a greater effect on DNA methylation with 176 differentially methylated regions. Genomic localization and annotation of DMRs with the closest gene revealed that ~78% overlapped a gene, whereas ~4% were in regions upstream or downstream of TSSs (Fig 4B). Calculations of Δ_me_ (the difference in average methylation between groups) for each DMR showed that DMRs were equally likely to be either hypo- or hyper-methylated with an average change in methylation between 10–30% (Fig 4C).

**Fig 5:**
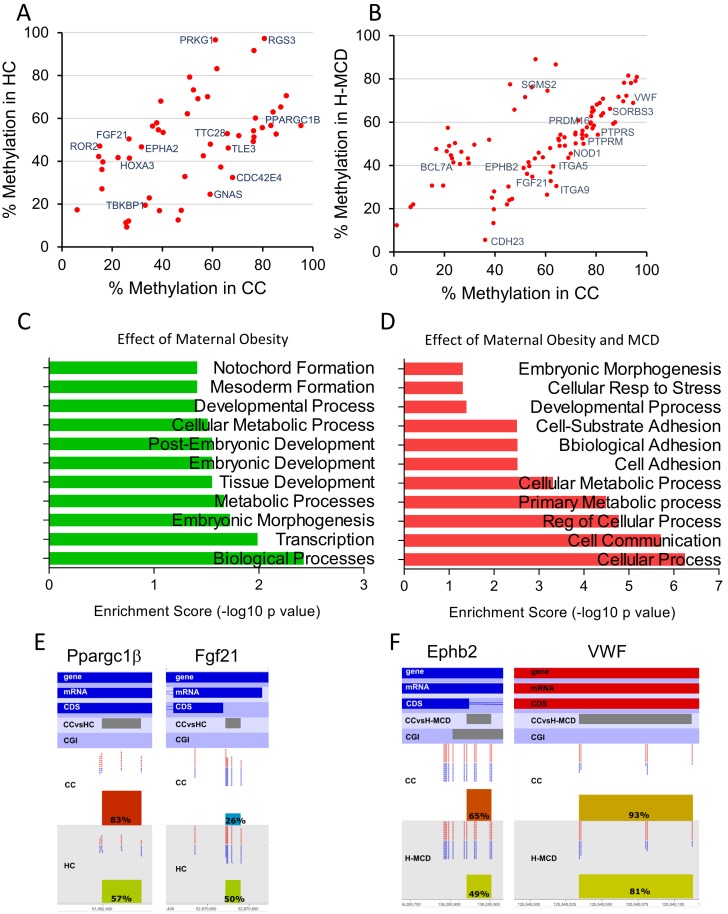
Changes in DNA methylation with either maternal HFD (HC vs CC) or maternal HFD and offspring MCD diet (H-MCD vs CC). Scatter plots of average methylation of DMRs showing altered methylation with **(A)** maternal HFD, or **(B)** the combination of maternal HFD and offspring MCD diet are presented along with annotation of key genes. **(C-D)** Enrichment of GO biological process terms of DMRs in proximity of the genes in **(C)** CC vs HC and **(D)** CC vs H-MCD comparisons. Maternal HFD influences methylation of regions proximal to **(E)** *Ppargc1β* and *Fgf21*. The combination of maternal HFD and offspring MCD diet feeding influences methylation of regions close to *Ephb2* and *Vwf*. Average methylation of the DMR is depicted on the histograms in the two lower tracks.

We next focused on interpreting changes in DNA methylation in two contexts, maternal HFD *per se* (HC vs CC, Fig 5A) and the combination of maternal HFD and offspring MCD diet (H-MCD vs CC, Fig 5B, *P<*0.0005 and Δ_me_ ≥10%). Enrichment of GO biological process terms of proximate genes revealed mainly developmental processes to be affected by maternal HFD (CC vs HC comparison) (Fig 5C). On the other hand, the combined effect of maternal HFD and offspring MCD diet mainly affected methylation of genes involved in cellular response to stress, cell substrate adhesion and cell adhesion (Fig 5D). Moreover, offspring of HFD dams had altered methylation patterns in DMRs of genes that play important roles in hepatic fibrosis and lipid accumulation, including *Ppargc1β*, *Fgf21*, *Ephb2* and *VWF* (Fig 5E and 5F). The complete list of DMRs is presented in S7 and S8 Tables. These findings suggest that maternal HFD is associated with epigenetic changes in pathways relevant to hepatic function and fibrosis.

### Maternal HF leads to distinct changes in the cecal microbiome of offspring

**Fig 6:**
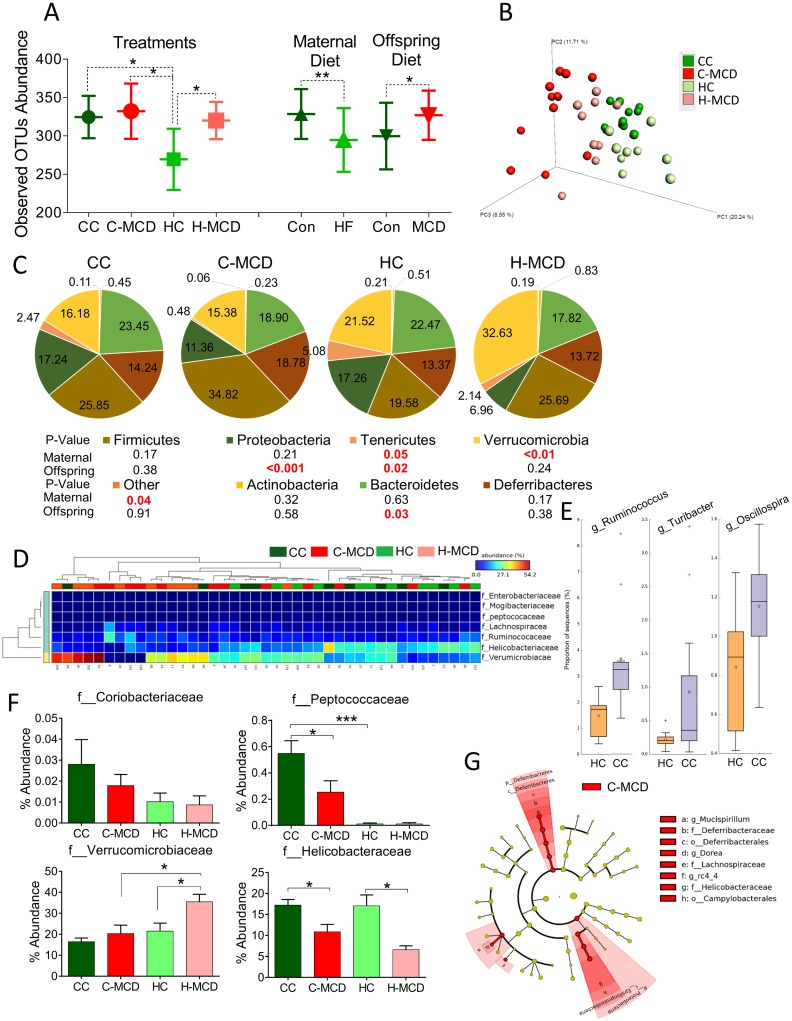
Effect of maternal HF and offspring MCD diet challenge on cecal microbiota. **(A)** α-diversity (OTU abundance) between treatments (CC, C-MCD, HC and H-MCD) or as main effects of maternal HFD and offspring MCD diet. **(B)** PCoA of gut microbiota composition based on unweighted UniFrac distances in offspring from control and HFD dams fed control or MCD diet (n = 9–11 mice per group) **(C)** Pie charts showing relative composition of gut microbial phyla in offspring groups. **(D)** Hierarchical clustering of family-level OTUs using STAMP showing predominant separation by offspring (MCD) diets. **(E)** Effect of maternal HFD on at least genera i.e. *Ruminococcus*, *Turibacter* and *Oscillospria*. **(F)** Abundance of specific microbial families in CC, C-MCD, HC and H-MCD groups (n = 9–11 mice per group). Data are expressed as means ± SE. Statistical differences in OTU abundance were determined using a one-way ANOVA followed by Student-Newman-Keuls post hoc analyses (*p<0.05, ***p<0.001). **(G)** Cladogram from LEfSe analysis showing taxa enriched in microbiota from mice fed MCD diets.

The gut microbiome represents another important mechanism linking maternal diet to offspring health. Therefore we examined changes in the microbial ecology in offspring from control and HFD-fed dams. α-diversity measures are indicative of phylogenetic species richness within a sample, while *β*-diversity represents compositional differences between samples. Maternal HFD significantly lowered α-diversity of gut microbiota (observed OTUs, CC = 324±27.6 vs HC = 269.5±39.9, p<0.05). On the other hand, MCD diet by itself irrespective maternal diet led to increased α-diversity (Con 299.78±43.45 vs MCD 327.06±32.11, p<0.05). Maternal HFD irrespective of offspring MCD diet led to reduced OTU numbers and less α-diversity (Con 328.62±32.50 vs HFD 294.75±41.59, p<0/01) (Fig 6A).

Principal coordinate analysis (PCoA) of unweighted UniFrac distances performed on the OTU abundance matrix showed that the *β*-diversity of gut microbial communities was significantly different between MCD and control diet-fed offspring as well as offspring from control and HFD dams (p<0.01) (Fig 6B). Offspring MCD treatment led to significant differences in *Tenericutes*, *Proteobacteria* and *Bacterioidetes* (p<0.05). Maternal HFD feeding led to increase in *Tenericutes*, *Verrucomicrobia* phyla (p<0.05) (Fig 6C). Clustering analysis using STAMP revealed that families predominantly grouped according to offspring diets (MCD) (Fig 6D). Genus level comparisons showed a significant effect of maternal obesity on at least three genera, i.e. *Ruminococcus*, *Turibacter* and *Oscillospria* (p<0.05) (Fig 6E). Consistent with STAMP analysis at the family level, *Helicobacteriaceae* was significantly reduced in MCD treated groups in offspring from both control and HFD-fed dams (Fig 6F) (p<0.01). *Coriobacteriaceae* and *Peptococcaceae* were reduced in offspring from HFD dams irrespective of offspring diet (Fig 6F). Linear discriminant analysis (LDA) of effect size indicated that the phylum *Deferribacteria* and *Proteobacteria* were enriched in MCD treated offspring of lean dams. (Fig 6G, S9 Table).

**Fig 7:**
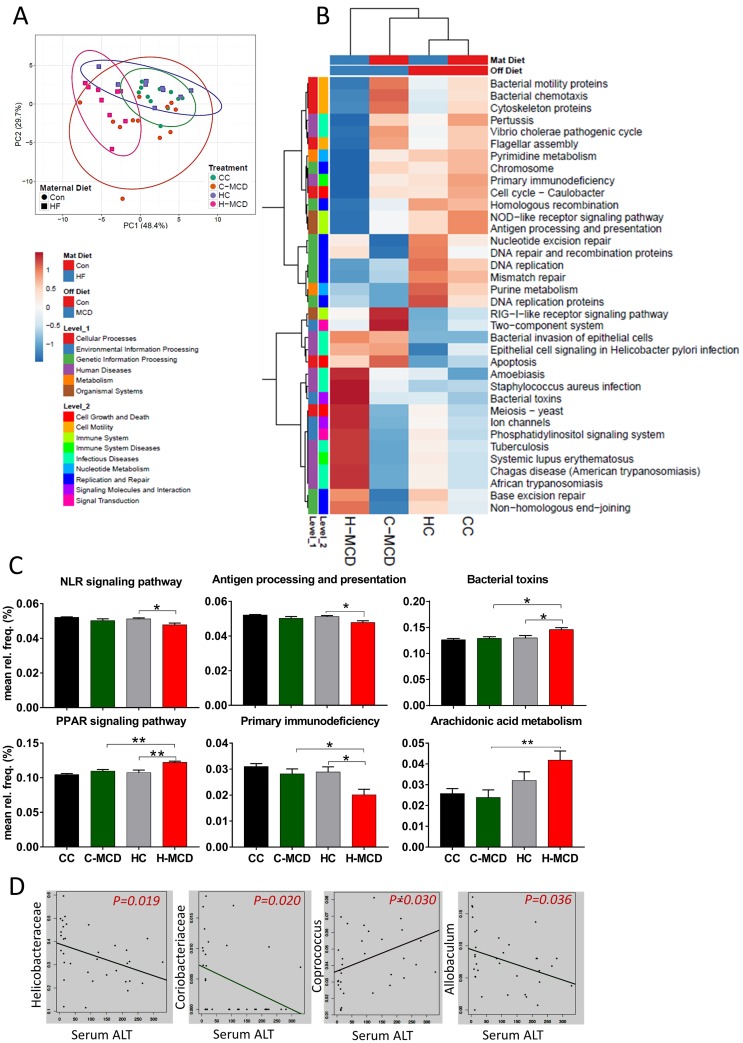
Maternal HF diet and offspring MCD diet alters cecal microbial functions. **(A)** Principal component analysis of predicted functional metagenomic pathways using PICRUSt **(B)** Heat map of PICRUSt predicted pathways shows maternal HFD affects microbial functions including nucleotide excision repair, glycerophospholipid metabolism and chloroalkane degradation. **(C)** MCD diet especially in HFD-dam offspring increases genes associated with bacterial toxin, epithelial cell signaling, primary immunodeficiency, arachidonic acid metabolism and bacterial invasion. **(D)** MaAslin analysis of ALT levels and OTU abundance in offspring shows significant correlation with *Helicobacteriaceae*,*Coriobacteriaceae*, *Coprococus* and *Allobaculum* families. Data are expressed as means ± SE. Statistical differences in predicted functional pathways were determined using a one-way ANOVA followed by Student-Newman-Keuls post hoc analyses (*p<0.05, ***p<0.01).

Prediction of functional composition of the microbiome was done using PICRUSt. Principal component analysis showed that the H-MCD offspring prominently separated from CC offspring (Fig 7A). Hierarchical clustering also showed greater main effect of MCD diets with genes involved in immune system, infectious diseases, nucleotide metabolism, signaling pathways regulating cell growth and motility (Fig 7B). Further examination showed that genes related to bacterial toxins, bacterial invasion of epithelial cells and apoptosis were increased in H-MCD offspring (Fig 7C). Finally, we examined associations between abundance of bacterial families and serum ALT levels among all offspring using Multivariate Association with Linear Models (MaAslin). These analyses identified *Helicobacteriaceae*, *Coriobacteriaceae*, *Coprococus* and *Allobaculum* families to be significantly correlated (p<0.05) with serum ALT levels (Fig 7D).

## Discussion

Over-nutrition leading to maternal obesity during pregnancy has profound influences on the risk of obesity and metabolic disease in the offspring. These include increased insulin resistance [3], hepatic steatosis and lipid biosynthesis [6,9,11,23,24], reduced skeletal muscle mass [25] and impairment of glucose homeostasis [26] in offspring. Using a rat model of controlled overfeeding, we have previously demonstrated that *in utero* exposure to maternal obesity leads to metabolic programming of the liver, characterized by fatty liver, metabolic inflexibility and altered lipid biosynthesis / oxidation and alterations in mitochondrial function [6,7,23]. In the present study we examined the consequences of maternal HFD exposure on offspring hepatic responses to well-established steatosis and inflammatory paradigms. Offspring from HFD dams: a) gain more weight when challenged with HFD and develop pro-inflammatory gene signatures, b) show alterations in DNA methylation amongst key developmental genes and c) have significant shifts in the gut microbiota composition.

The majority of previous studies in rodents have primarily focused on the body composition and cardiovascular consequences for the offspring following maternal HFD [27,28,29]. Using a model of HFD in mice, Mouralidarane et al. elegantly demonstrated the role of maternal diet in development of offspring NAFLD [30,31]. Likewise, Bruce et al., showed development of steatohepatitis in mice born to HFD-fed dams by 30 wk of age, associated with greater lipogenic genes and mitochondrial dysfunction [10]. Previous work from our group showed that *in utero* exposure to maternal obesity lead to disrupted circadian expression of metabolic genes in the liver [32], and was associated with metabolic inflexibility, steatosis and lipogenic gene expression in offspring [3,6,7,23]. Our findings are consistent with previous reports and further evaluate fibrogenic signaling following MCD diets which have not be examined previously. The present study provides unique mechanistic insights into transcriptome level changes associated with programming. Un-biased RNA-seq based transcriptomic profiling revealed alterations in immune response, fibrogenic and apoptosis related genes to be predominantly regulated in response to maternal HFD. Importantly, while high-fat diets are effective in leading to weight gain and steatosis, only modest increases in hepatic inflammatory response are typically observed. However even under these conditions, offspring of HFD-dams showed robust inflammatory signaling and greater steatosis in the liver. To more thoroughly examine the sensitivity of the offspring to NASH-like hepatic pathology we challenged offspring with a MCD diet. MCD diets produce steatosis due to impaired VLDL secretion and induces a severe inflammatory response [33]. To our knowledge this is the first report to assess offspring response to MCD diet in relation to maternal HFD. Clearly, maternal HFD worsened the offspring’s response to the MCD diet as evidenced by both histopathological and gene expression results. An important distinguishing feature of MCD challenge is that programmed effects of hepatic inflammatory and fibrogenic responses are uncoupled from body weight differences in the offspring. Genes related to fibrosis, apoptosis and inflammation were significantly up-regulated in MCD-treated offspring from HFD-dams compared to lean dams. These findings are consistent with previously observed increases in inflammatory and fibrogenic genes in rats and mice following MCD diet [34,35].

A salient finding from our work is that both maternal high-fat and offspring MCD diets influenced DNA methylation patterns in the offspring. Epigenetic modifications, such as methylation of cytosines and covalent post-translational modifications of histone tails have been widely hypothesized as molecular mechanisms linking complex gestational influences to offspring outcomes. Indeed, maternal obesity and dietary changes (such as low-protein and choline-deficient diets) have been shown to alter methylation of genes involved in development, metabolism, circadian rhythms [32,36], cell cycle [37] and other critical processes [38,39]. In the current study, genome-scale DNA methylation analysis of livers showed altered methylation of genes associated with key pathways such as cell adhesion and cell communication. The combination of maternal HFD and MCD diet altered DNA methylation of *EphB2* and *VWF*, whereas maternal HFD alone changed methylation of *HNF4A*, *Ppargc1β* and *Fgf21*. *EphB2* has been recently shown to modulate hepatic fibrosis in several contexts [40] [41]. Likewise, *VWF* is associated with liver disease as *VWF* antigen levels were elevated in plasma from cirrhotic and acute liver failure patients [42]. Yilmaz et al reported increased serum *Fgf21* levels in patients with NAFLD and suggest that *Fgf21* may be an independent predictor of hepatic steatosis [43]. Our data suggest that maternal HFD is associated with epigenetic alterations in *EphB2*, *VWF*, *Ppargc1β* and *Fgf21* that may contribute to the pathogenesis of NASH. While the present work presents a definitive comprehensive catalog of epigenetic changes associated with maternal HFD and offspring MCD diets, much work is warranted in understanding the mechanistic relationship between these epigenetic changes and underlying pathways promoting susceptibility.

A second important mechanism examined in the present studies was alterations in the offspring microbiome. Most studies examining developmental programming have focused on intrauterine and placental mechanisms contributing to offspring phenotypes. Nonetheless, in a majority of models including the human scenario, exposure to maternal nutrition and diet indirectly continues throughout lactation. Hence, an important avenue of persistent changes in the offspring may be mediated via disruption of the gut microbiome. The gut microbiota has a close anatomical and functional relationship with the liver via the portal circulation [44]. Dysbiosis in microbiome can increase gut permeability to bacterial products contributing to hepatic inflammation and fibrosis. Hence, we assessed if maternal HFD and offspring MCD diets influenced microbial profiles. In addition, the gut microbiome composition during the infant stage of life correlates with later microbiome colonization, indicating that dysbiosis in infancy due to maternal obesity exposure may have later consequences on metabolic health [45]. In the current study, fecal species richness is diminished in offspring of dams fed HFD with both maternal and offspring HFD showing a significant influence. Consistent with findings from the current study, Zhu et al. showed fecal species richness was diminished in obese subjects and NASH patients compared to controls and most samples clustered by health status but not by age, gender, or ethnicity, indicating a specific connection between the liver phenotype and gut microbiome [46]. Indeed, a recent report by Lemas at el., also suggest that early infant microbiome is influenced by maternal diet, obesity status and milk composition [47]. Similar persistent changes in gut microbiome dysbiosis have also be observed in non-human primate models of maternal HFD [36]. While our studies do not provide evidence for causality, they add to the growing consensus of findings showing early programming of gut microbial configurations may contribute to diverse health offspring outcomes including NAFLD and NASH.

## Conclusion

We have demonstrated that maternal HFD alters the response to post-weaning HFD in the offspring including hyper-responsiveness in weight gain and fatty liver associated with obesity. Offspring from HFD-dams develop unique transcriptomic changes especially in response to post-weaning HFD, typified by greater hepatic immune response. Maternal HFD-induced alterations are associated with increased expression of classic pro-fibrogenic genes in the liver following MCD diets. Consistent with the premise that *in utero* programming leads to epigenetic changes, offspring of HFD-dams show alterations in DNA methylation of predominantly development-related genes. Further, previously unrecognized epigenetic changes associated with MCD diet exposure in the liver were identified, including hypomethylation of key regulators of cell adhesion. Finally, these studies identified novel changes in the richness and abundance of specific bacterial taxa associated with maternal diet. Changes in gut bacteria were influenced further by offspring MCD diet and correlated with extent of liver injury and alterations in microbial genes regulating bacterial invasion and toxins. These results suggest that maternal HFD and obesity are likely to predispose offspring to hepatic inflammation and fibrogenesis contributing to the pathogenesis of NASH.

## Supporting information

S1 Fig Experimental design, body weight, composition, litter size of dams and offspring. **(A)** Schematic representation of the experimental design. **(B)** Body weights of female C57BL/6J mice fed control or HFD for 12 wk starting at 5 wk of age. **(C)** Body composition analysis shows higher fat (%) and lower lean mass (%) in HFD females. **(D)** Litter size of control and HF diet fed dams **(E)** Body weight of male (con and HF n = 10 each) and female (con and HF n = 11 each) offspring from PND 8 to PND 28. **(F)** Body composition of offspring PND 126 (CC, CH, HC and HH). Data are expressed as means ± SE. Statistical differences are determined using a Student’s t-test. *p<0.05, **p<0.01 comparing control to HF diet fed mice.
(TIF)

S2 Fig Experimental design for MCD diet challenge. Experimental design showing examination of interactions between maternal HFD and offspring MCD diets. Offspring from dams fed control or HF diet were weaned at 4 wk. Starting at 7 wk of age offspring we challenged with methionine choline sufficient (CC and HC) and MCD diets for 25 days.
(TIF)

S1 Table Primers sequences used for real-time PCR. (XLSX)

S2 Table Serum metabolites (cholesterol, glucose, NEFA,Triglycerides) in offspring. (XLSX)

S3 Table Union of differentially expressed genes in either comparison (RNAseq data). (XLSX)

S4 Table mRNA expression of genes in livers of offspring (real-time PCR). (XLSX)

S5 Table mRNA expression of genes in con and HFD offspring following MCD diets. (XLSX)

S6 Table Circulating serum cytokine concentrations in MCD diet fed offspring from control and HFD fed dams. (XLSX)

S7 Table Differentially Methylated Regions (DMR) influenced by maternal HFD feeding. (XLSX)

S8 Table Differentially Methylated Regions (DMR) influenced by maternal HFD and MCD diet feeding in offspring. (XLSX)

S9 Table Microbial abundance (at genus level) in cecal contents of offspring. (XLSX)

S1 File Supplementary methods. (DOCX)
