# Supplementary figures and images for: Enhanced offspring predisposition to steatohepatitis with maternal high-fat diet is associated with epigenetic and microbiome alterations
Source: PLoS One. 2017 Apr 17;12(4):e0175675. doi: 10.1371/journal.pone.0175675 (PMC5393586; doi:10.1371/journal.pone.0175675)

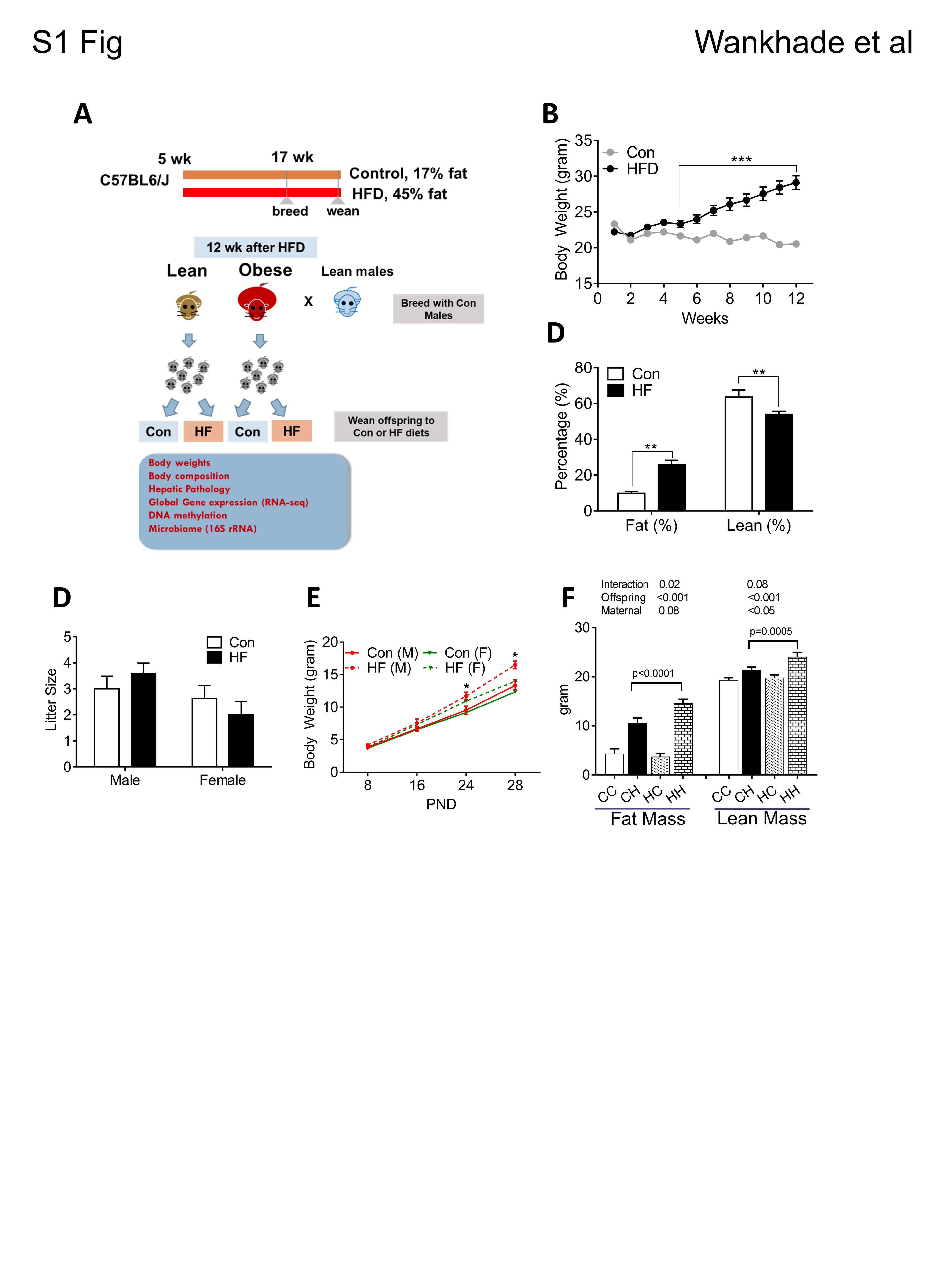

Supplement: S1 Fig — (A) Schematic representation of the experimental design. (B) Body weights of female C57BL/6J mice fed control or HFD for 12 wk starting at 5 wk of age. (C) Body composition analysis shows higher fat (%) and lower lean mass (%) in HFD females. (D) Litter size of control and HF diet fed dams (E) Body weight of male (con and HF n = 10 each) and female (con and HF n = 11 each) offspring from PND 8 to PND 28. (F) Body composition of offspring PND 126 (CC, CH, HC and HH). Data are expressed as means ± SE. Statistical differences are determined using a Student’s t-test. *p<0.05, **p<0.01 comparing control to HF diet fed mice. (TIF) [file pone.0175675.s001.tif]

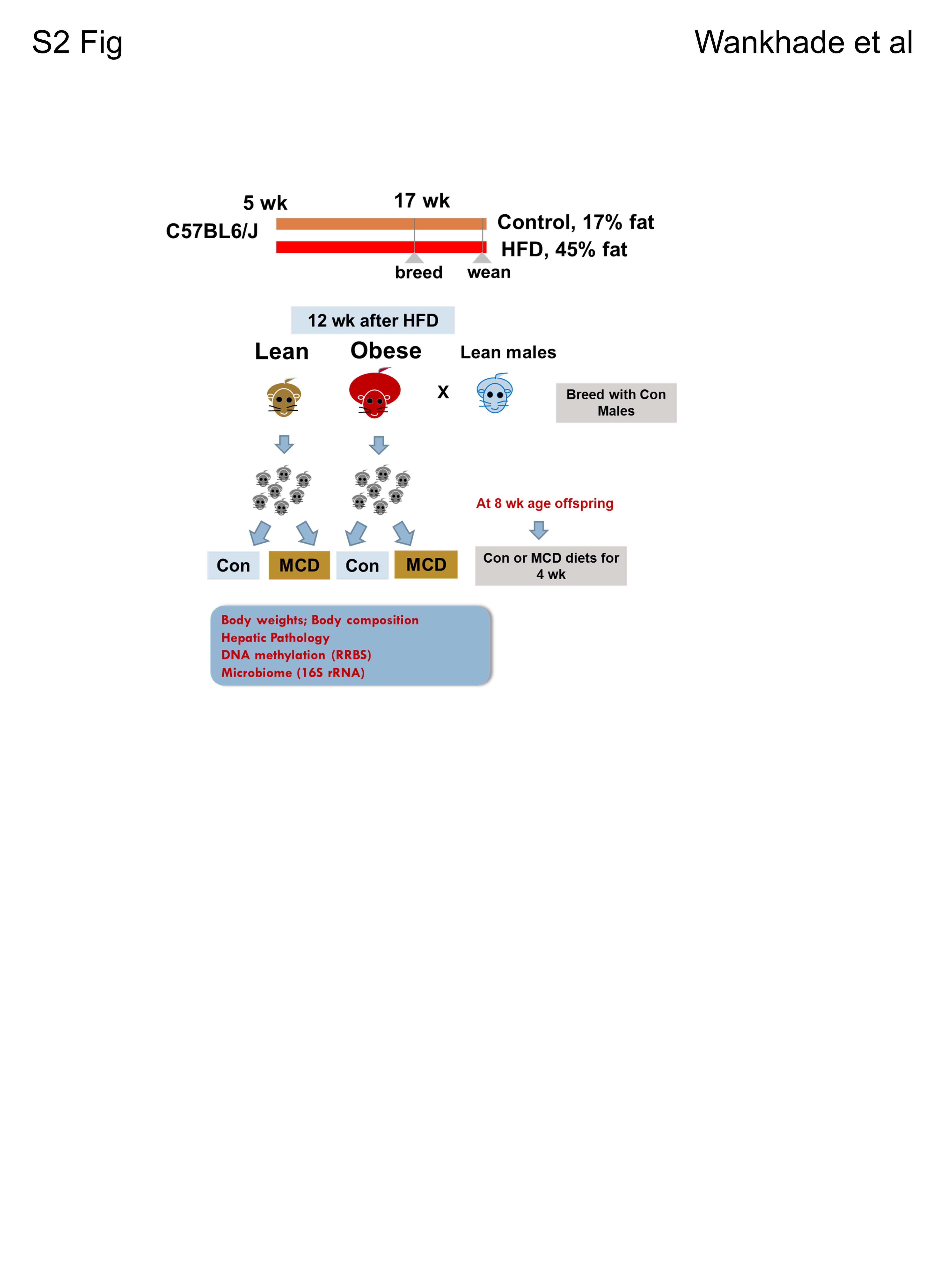

Supplement: S2 Fig — Experimental design showing examination of interactions between maternal HFD and offspring MCD diets. Offspring from dams fed control or HF diet were weaned at 4 wk. Starting at 7 wk of age offspring we challenged with methionine choline sufficient (CC and HC) and MCD diets for 25 days. (TIF) [file pone.0175675.s002.tif]
