# Supplementary material for: Enhanced offspring predisposition to steatohepatitis with maternal high-fat diet is associated with epigenetic and microbiome alterations
Source: PLoS One. 2017 Apr 17;12(4):e0175675. doi: 10.1371/journal.pone.0175675 (PMC5393586; doi:10.1371/journal.pone.0175675)
Supplement: S1 File — (DOCX) [file pone.0175675.s012.docx]

**Supplemental Methods**

***Experimental Design***

Female C57BL6/J mice (stock# 0664) were obtained from Jackson Laboratories (Bar Harbor, ME). Animals were housed in an AAALAC-approved animal facility in temperature and light controlled rooms (12h light-12h dark cycle). The Institutional Animal Care and Use Committee at the University of Arkansas for Medical Sciences approved all experimental protocols. Female mice at 4 weeks of age (n=10) were group housed (5 mice per cage) and acclimatized for 1 week. Starting at 5 weeks of age, all female mice were given *ad libitum* access to control (17% fat Harlan Teklad, TD#95095) or high-fat diets (HF, 45% fat, TD#08811) for 12 weeks. At 17 weeks of age females were bred with lean male mice (fed control diets throughout). Body weights of females were monitored weekly throughout and body composition was assessed non-invasively via QMR (EchoMRI) at 5 and 12 weeks of age. Upon birth, all offspring remained with birth dams until weaning and litter sizes were adjusted to 6 pups per litter around postnatal day 3 (PND3). Half of the offspring from each litter were randomly assigned to either control or HF diet at 4 weeks of age (**S1A Fig**). This experimental design led to four groups of offspring: viz. offspring born to control diet fed dams weaned onto Con (**CC**, n=10) or HFD (**CH**, n=11) and offspring born to HFD fed dams were weaned onto Con (**HC**, n=7) or HFD (**HH**, n=7). Since phenotypes of liver pathology (reported herein) were most prominent in males, only data from male offspring are presented. Post-weaning diet intervention was 14 weeks. Throughout the study, offspring body weight was monitored weekly. Offspring body composition was assessed at the start (5 wk) and at the end (12 wk) of the study. Offspring food intake was monitored three days a week throughout the 14-week diet challenge **(S1A Fig).** Offspring were euthanized with CO_2_. Blood was collected via cardiac puncture for serum separation to measure NEFA, triglycerides, cholesterol and glucose.

***Preparation of RNA-seq libraries***

Total RNA was isolated from 100 mg liver tissue using a combination of TRI reagent and RNeasy-mini columns (Qiagen, Valencia, CA), including on-column DNase digestion [2]. RNA quality and integrity was confirmed spectrophotometrically (A260/A280 ratio > 1.9) and via visualization using Experion RNA Std-Sens chips (BioRad, Hercules, CA). Equal amounts of polyA-mRNA from 2–3 mice were pooled, to generate three biologically distinct replicates per group representing all animals (CC n=10, CH n=11, HC n=7, HH n=7). Poly-A RNA was isolated from 5 μg of total RNA using Dynabeads^®^ mRNA-Direct kit (Invitrogen, Carlsbad, CA) and procedures described previously [2]. Briefly, poly-A RNA was captured by addition of 100 μl of Oligo-(dt)_25_ Dynabeads in 150 μl of lysis buffer. The mixture was incubated on a rotary shaker for 20 min at room temperature. mRNA-bead complexes were washed twice with 100 μl of wash buffer A (10 mM Tris-HCl, pH 7.5, 0.15 M LiCl, 1 mM EDTA, 0.1% LiDS), followed by two washes (100 μl each) with wash buffer B (10 mM Tris-HCl, pH 7.5, 0.15 M LiCl, 1 mM EDTA). RNA was eluted from the beads in 11 μl of nuclease free water by heating to 65 °C for 5 min.

Following purification, mRNA was sheared to ~150 bp fragments using a Covaris S2 instrument (120 μl volume, 10% DC, 5 intensity, 200 cpb, 7.5 min). Fragmented poly-A RNA was precipitated using sodium acetate/ethanol and reconstituted in 14 μl of nuclease free water. RNA-seq library construction was carried out using NEB-Next reagents (New England Biolabs, Ipswich, MA). First and second strand cDNA synthesis, end-filling using Klenow fragment, and dA-tailing were carried out using manufacturer’s recommendations. Ligation with Illumina’s paired-end adapters for multiplexed sequencing was performed with 1 μl of T4 DNA ligase, 0.3 μM of annealed adapters, in a 50 μl reaction volume for 30 min at room temperature. Ligated products were separated using a high-resolution 2% agarose gel, and products around 200 bp (±50 bp) were excised and purified using Qiagen gel extraction kit (Qiagen, Valencia, CA). Size-selected cDNA libraries were amplified using indexed primers. PCR was carried out for 12-14 cycles using 29 μl of template, 1 μl of forward and reverse primers (25 μM), and 1 U Phusion high-fidelity DNA polymerase (New England Biolabs). PCR products were purified using Qiaquick PCR purification columns (Qiagen, Valencia, CA) and eluted in 30 μl final volume. A small aliquot (~1 μl) was evaluated using DNA1K chip (Experion, Bio-Rad, Hercules, CA) to confirm the absence of primer-dimers and other spurious products. Quantification of the RNA-seq libraries was done via Qubit dsDNA HS Assay kit.

**RNA-seq analysis**

RNA-seq analysis was carried out using the Tuxedo pipeline (TopHat-Cufflinks-CummeRbund) [4]. High quality reads were aligned using TopHat using default options and resulting BAM files were utilized in Cufflinks to produce a transcriptome assembly. Differentially expressed genes were identified against a merged transcriptome assembly generated via the cuffmerge function. Resulting outputs were analyzed in CummeRbund package in R-Bioconductor. FPKM (fragments per kilobase per million mapped reads) values represent counts of reads mapping to a feature (gene, exon, etc.) normalized to both the overall sequencing coverage and the size of the feature. Differentially expressed genes between groups were identified using cuffdiff based on *P* value ≤ 0.05 and a minimum fold change of ± 2-fold (in pair-wise comparisons). Corrections for multiple testing were performed using the false discovery rate method [5]. Distribution of reads over genomic features was calculated using RSeQC from aligned data. Venn diagrams were generated in R [6]. Enrichment of gene ontology (GO) terms for biological/ molecular function performed using BiNGO using FDR corrected *p*-value < 0.01 [7].

***Genome-scale DNA methylation via RRBS***

DNA methylation changes associated with maternal and offspring diets were assessed using RRBS, which involves sequencing of bisulfite-converted *MspI* fragment libraries. Liver genomic DNA from offspring was isolated using a combination of proteinase-K digestion and Purelink genomic DNA isolation kits (Life Technologies). Three biologically distinct pools of genomic DNA (containing n=3 in each pool) were utilized to generate libraries. RRBS libraries were prepared as described by Gu *et al* [1]. Briefly, genomic DNA (0.5 μg) was digested with 200 U MspI and purified for end-repair, dA-tailing and overnight ligation with methylated adapters (Illumina). Ligated fragments ranging from 170 - 350 bp (representing 40-220 bp *MspI* fragments) were gel-excised, purified and utilized for two rounds of bisulfite conversion (total 14 h) using EpiTect Bisulfite kit (Qiagen). Purified DNA was amplified using barcoded primers suitable for Illumina sequencing, and *pfu* Turbo Hotstart DNA Polymerase (Agilent). PCR products were cleaned using AMPureXP beads (Agencourt). Libraries were quantified using Qubit dsDNA HS Assay kit (Invitrogen) and sequenced using a Nextseq 500 (75-bp, single end).

Reads were trimmed for adapter sequences using Trim Galore and filtered for quality score. Alignment and methylation calling were performed using Bismark [2,3]. Data analysis and summarization were done using SeqMonk and the DSS package in R [4]. The DSS package employs a Bayesian hierarchical model to estimate and shrink CpG-level dispersions using β-binomial distribution of methylation data. Biological variability is captured in the dispersion parameter and group differences are assessed at each CpG site. Only CpGs with minimum 5X coverage were included in the analysis. Comparisons between different groups (CC, C-MCD, HC and H-MCD) were performed using Wald test, and *P* values were adjusted for multiple testing using the FDR method [4] CpGs with *p* < 0.0001 (approximately corresponding to *q* value of 0.1) and a minimum difference in methylation (Δ_me_) of 10% were retained. These differentially methylated regions (DMRs) were annotated with the closest/overlapping transcription start sites (TSSs) (±10 kb) using Seqmonk and genomic location analysis was performed using CisGenome. Second, we examined whether maternal HFD and offspring diets affected methylation of promoters and CpG islands (CGI). Promoters were sub-classified into those overlapping or devoid of CGIs. The model-based CGI map described by Wu et al [5] was utilized. Frequency distribution of methylation status of features was computed. Statistical differences between groups were analyzed using χ^2^ test [3]. The lists of differentially expressed genes in relation to location of DMRs were analyzed for GO biological process and molecular function enrichment using the BiNGO plugin in Cytoscape [6].

***Microbial community profiling of cecal contents via 16S rRNA amplicon sequencing***

Bacterial DNA was isolated from cecal contents using QIAamp Fast DNA stool mini kit (Qiagen) including a bead-beating step. Fifty nanograms of genomic DNA was utilized for amplification of the V4 variable region of the 16S rRNA gene using 515F/806R primers. Forward and reverse primers were barcoded as described by Kozich et al [7] to accommodate multiplexing up to 384 samples per run. Paired-end sequencing (2 X 250 bp) of pooled amplicons was carried out using Illumina Miseq platform [8] with ~30% PhiX DNA.

Processing and quality filtering of reads was performed by using scripts in QIIME (v1.9.1) [9] and other in-house and published scripts. Paired reads were stitched with PEAR, an over-lapping paired-end reads merger algorithm which evaluates all possible paired-end read overlaps minimizing false positive hits [10]. Reads were further filtered based on Phred quality scores and for chimeric reads using USEARCH61 [11]. Filtered reads were demultiplexed within QIIME and samples with less than 5000 reads were excluded from further analysis. UCLUST was used to cluster sequences into operational taxonomical units (OTUs based on >97% identity) [11]. OTU picking was performed using an open-reference method which encompasses clustering of reads against a reference sequence collection and also performs de novo OTU picking on the reads which fail to align to any known reference sequence in the database [12]. To eliminate erroneous mislabeling, the resulting OTU tables were checked for mislabeling sequences [13]. Representative sequences were further aligned using PyNAST with the Greengenes core-set alignment template [14]. Construction of the phylogenetic tree was performed using the default (FASTTREE) method in QIIME [15]. Alpha rarefaction was performed using the phylogenetic diversity, Chao1 and observed species metrics. Beta diversity estimation was carried out by computing weighted and un-weighted UniFrac distances between samples using QIIME [16]. All samples were clustered based on their between-sample distances using UPGMA, and subsequent jack-knifing was performed by resampling methods. Comparisons of intergroup and intragroup diversity were performed using ANOVA including correction for multiple comparisons. Differences in OTU abundance between groups were identified using STAMP [17] and visualized using Clustvis. Phylogenetic Investigation of Communities by Reconstruction of Unobserved States (PiCRUST) was used to identify differences in predictive metagenome function [18]. OTUs identified using a closed-reference procedure [14], were normalized by the predicted 16S copy number, and functions were predicted using the GreenGenes 13_5 database for KEGG Orthologs. From this, a BIOM table containing the predicted metagenome for each sample was generated. Downstream statistical analysis was performed using STAMP [17].

We also examined group difference using LefSe which utilizes Linear Discriminant Analysis of Effect Size. Associations of OTU abundance with specific phenotypic parameters (serum ALT) was performed using MaAsLin which is a multivariate statistical framework that performs boosted, additive general linear models between metadata and OTU abundance. Analysis for LefSe and MaAsLin were carried out using the default setting on Galaxy [19].

**References:**

1. Delidaki M, Gu M, Hein A, Vatish M, Grammatopoulos DK (2011) Interplay of cAMP and MAPK pathways in hCG secretion and fusogenic gene expression in a trophoblast cell line. Mol Cell Endocrinol 332: 213-220.

2. Krueger F, Andrews SR (2011) Bismark: a flexible aligner and methylation caller for Bisulfite-Seq applications. Bioinformatics 27: 1571-1572.

3. Smallwood SA, Tomizawa S, Krueger F, Ruf N, Carli N, et al. (2011) Dynamic CpG island methylation landscape in oocytes and preimplantation embryos. Nat Genet 43: 811-814.

4. Wu H, Xu T, Feng H, Chen L, Li B, et al. (2015) Detection of differentially methylated regions from whole-genome bisulfite sequencing data without replicates. Nucleic Acids Res 43: e141.

5. Wu H, Caffo B, Jaffee HA, Irizarry RA, Feinberg AP (2010) Redefining CpG islands using hidden Markov models. Biostatistics 11: 499-514.

6. Maere S, Heymans K, Kuiper M (2005) BiNGO: a Cytoscape plugin to assess overrepresentation of gene ontology categories in biological networks. Bioinformatics 21: 3448-3449.

7. Kozich JJ, Westcott SL, Baxter NT, Highlander SK, Schloss PD (2013) Development of a dual-index sequencing strategy and curation pipeline for analyzing amplicon sequence data on the MiSeq Illumina sequencing platform. Appl Environ Microbiol 79: 5112-5120.

8. Bokulich NA, Subramanian S, Faith JJ, Gevers D, Gordon JI, et al. (2013) Quality-filtering vastly improves diversity estimates from Illumina amplicon sequencing. Nat Methods 10: 57-59.

9. Caporaso JG, Kuczynski J, Stombaugh J, Bittinger K, Bushman FD, et al. (2010) QIIME allows analysis of high-throughput community sequencing data. Nat Methods 7: 335-336.

10. Zhang J, Kobert K, Flouri T, Stamatakis A (2014) PEAR: a fast and accurate Illumina Paired-End reAd mergeR. Bioinformatics 30: 614-620.

11. Edgar RC (2010) Search and clustering orders of magnitude faster than BLAST. Bioinformatics 26: 2460-2461.

12. Rideout JR, He Y, Navas-Molina JA, Walters WA, Ursell LK, et al. (2014) Subsampled open-reference clustering creates consistent, comprehensive OTU definitions and scales to billions of sequences. PeerJ 2: e545.

13. Knights D, Kuczynski J, Charlson ES, Zaneveld J, Mozer MC, et al. (2011) Bayesian community-wide culture-independent microbial source tracking. Nat Methods 8: 761-763.

14. McDonald D, Price MN, Goodrich J, Nawrocki EP, DeSantis TZ, et al. (2012) An improved Greengenes taxonomy with explicit ranks for ecological and evolutionary analyses of bacteria and archaea. ISME J 6: 610-618.

15. Price MN, Dehal PS, Arkin AP (2010) FastTree 2--approximately maximum-likelihood trees for large alignments. PLoS One 5: e9490.

16. Lozupone C, Knight R (2005) UniFrac: a new phylogenetic method for comparing microbial communities. Appl Environ Microbiol 71: 8228-8235.

17. Parks DH, Tyson GW, Hugenholtz P, Beiko RG (2014) STAMP: statistical analysis of taxonomic and functional profiles. Bioinformatics 30: 3123-3124.

18. Langille MG, Zaneveld J, Caporaso JG, McDonald D, Knights D, et al. (2013) Predictive functional profiling of microbial communities using 16S rRNA marker gene sequences. Nat Biotechnol 31: 814-821.

19. Segata N, Izard J, Waldron L, Gevers D, Miropolsky L, et al. (2011) Metagenomic biomarker discovery and explanation. Genome Biol 12: R60.
